# Wearable Technology for High-Frequency Cognitive and Mood Assessment in Major Depressive Disorder: Longitudinal Observational Study

**DOI:** 10.2196/12814

**Published:** 2019-11-18

**Authors:** Francesca Cormack, Maggie McCue, Nick Taptiklis, Caroline Skirrow, Emilie Glazer, Elli Panagopoulos, Tempest A van Schaik, Ben Fehnert, James King, Jennifer H Barnett

**Affiliations:** 1 Cambridge Cognition Cambridge United Kingdom; 2 Cognition Kit Cambridge United Kingdom; 3 Takeda Pharmaceuticals USA Deerfield, IL United States; 4 School of Psychological Science, University of Bristol Bristol United Kingdom; 5 Ctrl Group London United Kingdom; 6 Department of Psychiatry University of Cambridge Cambridge United Kingdom

**Keywords:** depression, cognition, mood, mobile health, mHealth, mobile apps, ecological momentary assessment, digital phenotyping, digital biomarkers

## Abstract

**Background:**

Cognitive symptoms are common in major depressive disorder and may help to identify patients who need treatment or who are not experiencing adequate treatment response. Digital tools providing real-time data assessing cognitive function could help support patient treatment and remediation of cognitive and mood symptoms.

**Objective:**

The aim of this study was to examine feasibility and validity of a wearable high-frequency cognitive and mood assessment app over 6 weeks, corresponding to when antidepressant pharmacotherapy begins to show efficacy.

**Methods:**

A total of 30 patients (aged 19-63 years; 19 women) with mild-to-moderate depression participated in the study. The new Cognition Kit app was delivered via the Apple Watch, providing a high-resolution touch screen display for task presentation and logging responses. Cognition was assessed by the n-back task up to 3 times daily and depressed mood by 3 short questions once daily. Adherence was defined as participants completing at least 1 assessment daily. Selected tests sensitive to depression from the Cambridge Neuropsychological Test Automated Battery and validated questionnaires of depression symptom severity were administered on 3 occasions (weeks 1, 3, and 6). Exploratory analyses examined the relationship between mood and cognitive measures acquired in low- and high-frequency assessment.

**Results:**

Adherence was excellent for mood and cognitive assessments (95% and 96%, respectively), did not deteriorate over time, and was not influenced by depression symptom severity or cognitive function at study onset. Analyses examining the relationship between high-frequency cognitive and mood assessment and validated measures showed good correspondence. Daily mood assessments correlated moderately with validated depression questionnaires (r=0.45-0.69 for total daily mood score), and daily cognitive assessments correlated moderately with validated cognitive tests sensitive to depression (r=0.37-0.50 for mean n-back).

**Conclusions:**

This study supports the feasibility and validity of high-frequency assessment of cognition and mood using wearable devices over an extended period in patients with major depressive disorder.

## Introduction

Major depressive disorder (MDD) is characterized by symptoms of low mood, diminished interest and pleasure in daily activities, feelings of worthlessness or guilt, fatigue, sleeping and appetite disturbances, and thoughts of death or suicide. MDD is a leading cause of disease burden and disability worldwide [1,2]. Cognitive symptoms, including difficulty concentrating or making decisions, are features of MDD [3] that may offer a target for intervention [4].

Cognitive symptoms of MDD include deficits in several domains, including processing speed, attention, executive function, learning, and memory [5-7]. Cognitive symptoms are seen in first-episode depression [6,8], persist beyond the symptoms of low mood [9-11], contribute to the risk of relapse [12], and worsen with repeated depressive episodes [13,14].

Cognitive MDD symptoms contribute to disability burden [15]. Poorer memory [15,16], attention, and executive function [17] have been associated with impairment in activities of daily living. Cognitive symptoms have also been associated with poor occupational functioning [18] and unemployment [19], work-related disability, and adverse psychosocial outcomes [20-22]. Longitudinally, improved cognitive function has been associated with higher rates of employment at follow-up in a variety of psychiatric illnesses, including MDD [23]. Treating these symptoms has the potential to improve functional outcomes and quality of life.

Research has highlighted discrepancies between objectively measured cognitive function and patients’ self-report from questionnaires, with the latter being affected by depressed mood [15,24,25]. This inconsistency highlights the need for subjective and objective data to be acquired to provide accurate clinical information. A key obstacle is the lack of readily available tools for cognitive assessments outside the clinic. Such tools could support the treatment and remediation of cognitive symptoms associated with MDD.

Mobile digital technologies allow for sampling outside of the clinic and in the patient’s home or work environment, providing a shared platform for clinicians and patients to monitor symptoms [26]. In depression, mobile apps have tracked changes in patient-reported mood [27-29] and have been used as part of randomized controlled trials to evaluate treatment efficacy [30]. However, these studies have relied on quantitative self-report or simple sensing and monitoring technologies [26].

This study examined feasibility, that is, viability of brief, high-frequency cognitive and mood assessment over an extended period of time (6 weeks) implemented on an Apple Watch app in individuals with MDD, and validity, defined as agreement between these high-frequency data and validated measures of mood and cognition. Coprimary endpoints were (1) adherence, examined separately for high-frequency cognitive and mood assessment and (2) correlations between daily measures of cognition with traditional full-length cognitive assessments, as specified in study details in the clinical trials registration [31].

The following secondary outcomes were examined, as described in the study analysis plan [32]: (1) the relationship between daily mood measures with full-length validated questionnaires and (2) the reliability of heart rate and activity sensors acquired via the Apple iPhone and Apple Watch (Apple Inc) apps. In addition, exploratory analyses examined the interrelationship of mood and cognitive measures acquired in low- and high-frequency assessment.

## Methods

### Participants and Recruitment

A recruitment target of 30 was set for this study, commensurate with usual practice for feasibility studies [33]. A sample size of 30 allows estimation of a compliance rate of 80%, with 95% CIs of ±12.8%. This sample size also provides 80% power to detect correlations of r=0.5.

A total of 556 adults underwent an initial screening for eligibility to participate in the study through a patient recruitment company with links to primary care providers and depression patient groups, to identify individuals with depression potentially suitable for the study. In total, 72 individuals were contacted for more detailed medical history information and to complete the Patient Health Questionnaire-9 (PHQ-9) [34] to obtain an index of depression severity. Participant eligibility was determined according to the following inclusion and exclusion criteria before study entry:

Inclusion criteria were primary psychiatric diagnosis of MDD; treated with antidepressant monotherapy; mild-to-moderate depression, defined by PHQ-9 scores between 5 and 15; aged 18 to 65 years; able to read and understand English; and owning their own iPhone.

Exclusion criteria were personal history of other psychiatric disorder (except nonprimary concurrent anxiety); manic or hypomanic episode; mental retardation, organic mental disorders, or mental disorders owing to a general medical condition as defined in the *Diagnostic and Statistical Manual of Mental Disorders 5th Edition*; neurological or neurodegenerative disorder; alcohol or other substance abuse or dependence (excluding nicotine or caffeine); responding only to combination or augmentation therapy in the current episode; hospitalization for MDD in 3 months or suicide attempt in 6 months before screening (or the participant was considered to be at significant risk of suicide or hospitalization); having received any investigational compound within 30 days before screening or 5 half-lives before screening, whichever is longer; concurrent participation in other clinical studies; or participation in 2 or more interventional studies in the year before screening.

In total, 30 of the 72 screened individuals were recruited into the study. Of the remaining screened individuals, 7 were eligible but not recruited. Others were excluded because of lack of an iPhone (n=4), insufficient time on medication (n=18), lack of antidepressant medication treatment history (n=2), polypharmacy (n=3), other psychiatric diagnosis or neurological condition (n=5), PHQ-9 higher than 15 (n=1), or insufficient information obtained in screening (n=2).

### Procedure

The study began with a visit to the study site and a short semistructured interview to explore each participant’s expectations and motivations for taking part. Researchers provided study hardware (an Apple Watch Series 2, paired with the participant’s own iPhone), presented the tasks, and gave participants the opportunity to practice using the tasks and device and ask questions. Participants were given contact details for the study center, where they could get in touch by email or phone if they experienced technical issues or had questions or concerns regarding their participation. Testing was completed in the subsequent 6 weeks (42 days), corresponding to the time when antidepressant pharmacotherapy shows efficacy in treating the mood symptoms of MDD. Participants were encouraged to respond to cognitive assessment wherever possible but not to worry when individual assessments were missed.

Data collected on the Apple Watch and iPhone were transferred automatically through Wi-Fi or data roaming via the participant’s iPhone to a secure data center held on Amazon’s Web service. This service provided identity and access control mechanisms to ensure participants (and only participants) had write access, and study managers only had read access. Where data for individual participants were not uploaded for 4 days, the research team made contact to ensure that the study equipment was working and to gain a better understanding of why assessments were not completed.

Full-length cognitive and validated self-report assessments were completed via a Web-based testing interface. Familiarization with the tests was completed during in-person assessments on the first day of participation. Full assessments were completed on 3 occasions: week 1 (between days 1 and 2), week 3 (days 18-24), and week 6 (days 40-46). Participants were sent a unique link to a secure Web page that delivered the test. On completion of assessment, and when the device established an internet connection, data were transferred to a secure Health Insurance Portability and Accountability Act of 1996 – compliant data center in the United States.

The study was completed with a 90-min, semistructured qualitative interview during week 6 at participants’ homes. Interviews explored participants’ experiences of assessment with the wearable technology, changes in motivation and adherence, and contextual factors that might have contributed to those changes. Study hardware was returned at this time.

### Measures

#### Daily Mobile Digital Assessments

**Figure 1:**
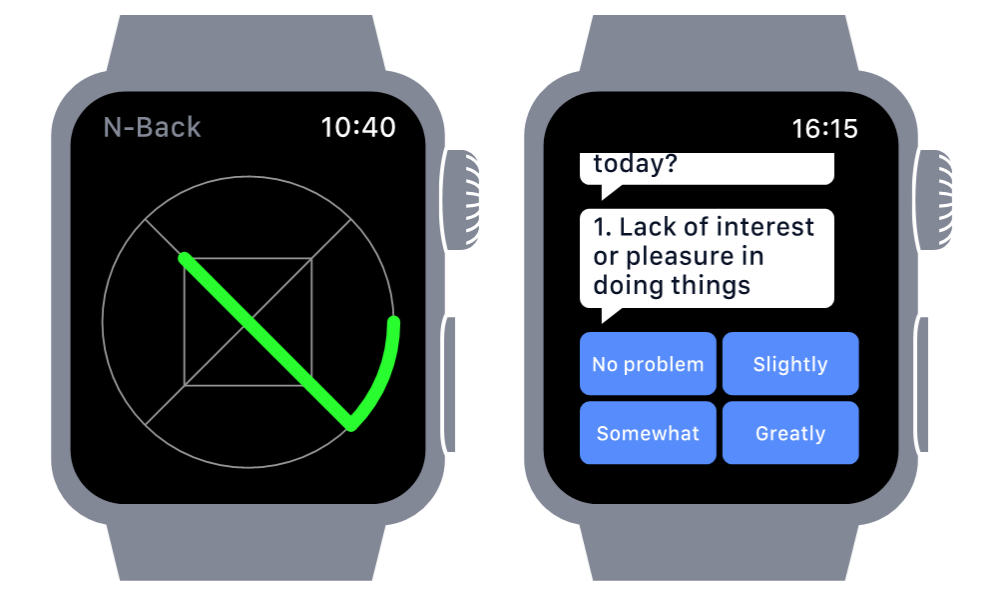
Symbol display for n-back (left) and mood assessment questions (right) presented on the Apple Watch. Participants were asked to tap the screen to respond to a match.

The Apple Watch provides a small touch screen for the presentation of stimuli and collection of participant responses and contains a range of sensors, including accelerometers and heart rate sensor. Participants were asked to wear the watch from 8 am to 10 pm for 6 weeks and to respond to assessment prompts. Additional step count data were acquired via the iPhone. An illustration of mood and cognitive assessment is provided in Figure 1.

#### High-Frequency Cognitive Assessments

Participants were prompted to complete cognitive assessments 3 times daily (morning, afternoon, and evening). Multiple prompts for cognitive testing were delivered to improve flexibility for participants unable to complete cognitive testing at specific points in the day and to yield data with the potential to examine diurnal changes (not examined in the current report).

Cognitive assessment was completed using a variant of the n-back task, a task which has shown sensitivity to impairments in MDD [35]. This variant was developed for brief high-frequency assessment after initial piloting indicated that a large pool of nonverbalizable stimuli were required to reduce ceiling effects over prolonged testing. A total of 9 symbols, randomly selected from a pool of 227, were presented for 600 ms 1 at a time over 30 trials. Participants were asked to respond when any symbol was the same as the symbol presented 2 trials previously. The primary outcome measure was dprime (the ratio of hits [correct detection of an n-back match] to false alarms [response during no match]). Each full assessment took 30 seconds to complete, after which participants were shown their n-back score.

#### High-Frequency Mood Assessments

Mood assessment was prompted up to twice daily (afternoon and evening). If participants completed the mood assessment in the afternoon, no prompt was delivered in the evening. Only 1 mood assessment was completed per day as participants were asked to reflect on and respond regarding their experiences over the past day.

Mood was assessed with 2 questions adapted from the PHQ-2, a validated brief form of the PHQ-9, which assesses only low mood and loss of interest or pleasure and is sensitive to depression and suitable for brief assessments [36]. One additional item assessing self-perceived concentration was taken from the Perceived Deficits Questionnaire—Depression (PDQ-D) [37,38], a measure that assesses subjective cognitive dysfunction in depression. Questions were modified from asking about symptom presence over multiple weeks to asking about symptoms over the past day. Wording was also shortened to facilitate presentation on a small screen.

Mood questions were presented in the following manner: *How much have the following problems bothered you over the past day?* Participants rated the following items: (1) lack of interest or pleasure in doing things; (2) feeling down, depressed, or hopeless; and (3) trouble concentrating on things (eg, newspaper, TV). Responses were coded on a 4-point scale of severity of symptoms (1=no problem, 2=slightly, 3=somewhat, 4=greatly). This scale was modified from the 4-point scale of the PHQ-9 to reflect within-day experiences and was kept consistent for the PDQ-D item.

#### Web-Based Full-Length Assessments

The Cambridge Neuropsychological Test Automated Battery (CANTAB) Connect Web-based testing interface was used to complete full-length cognitive testing and validated questionnaires on 3 occasions (weeks 1, 3, and 6). CANTAB cognitive assessments have shown sensitivity to a range of cognitive deficits in depression [10].

##### Cognitive Assessments

1. Spatial working memory (SWM) [39] examined participants’ ability to retain and manipulate visuospatial information and to strategize. Between 4 and 8 boxes were presented on the screen. Participants were asked to find tokens in the boxes and move them to a collection area and were instructed that they would not find a token in the same box twice in the same trial. Outcome measures included the following: (1) between errors, the number of times the participant revisited a box in which a token had been found (range of possible scores 0-175); and (2) strategy, the number of unique boxes from which a participant started a new search (range of possible scores 4-28). For both outcomes, lower scores indicated better performance.
2. The CANTAB rapid visual information processing (RVP) [40] test measured sustained attention and processing speed. Digits from 2 to 9 were presented successively at the rate of 100 digits per minute and in pseudorandom order. Participants were asked to respond to target sequences of digits (eg, 2-4-6, 3-5-7, 4-6-8). Two outcome measures were examined: (1) RVP A', a signal detection measure of sensitivity to the target regardless of response tendency (expected range is 0 to 1); and (2) RVP median latency of correct responses (maximum response time allowable 1800 ms).

##### Validated Questionnaires

1. The PHQ-9 [34] provided an index of depression severity, with higher scores reflecting greater symptom severity.
2. The PDQ-D [37] subscales of attention/concentration and planning/organization were summated to provide an index of participant-perceived cognitive symptoms. Higher scores reflect greater perceived cognitive symptoms.
3. The University of California Los Angeles Loneliness Scale (UCLA-LS) [41] measured subjective feelings of loneliness and social isolation. Higher scores reflect more severe loneliness and social isolation.

#### Semistructured Interviews

A copy of the discussion guides for semistructured interviews at study onset and end are provided in Multimedia Appendix 1.

### Statistical Analysis

#### High-Frequency Data Preparation and Cleaning

Adherence was assessed separately for cognitive function, mood reports, and activity. Adherence for mood and cognitive assessments was defined in line with methods described in the clinical trials registration [31]: each day was defined as adherent (with participants completing at least 1 full assessment each day) or nonadherent (days with no data). For Apple Watch activity and heart rate measures, nonwearing days (defined as days where <100 steps were recorded [42,43] [n=19 observations] or where heart rate was not recorded [n=6 additional observations]) were excluded from analyses. No minimum adherence was specified for participants to be included in analyses.

Percentage of adherent days was examined separately for mood, cognitive function, and activity for the duration of the study (defined as percentage of 42 days completed) and calculated for individual study weeks (weeks 1-6). In addition, for cognitive assessments, where responses were prompted 3 times daily, percentage of responses to all possible assessments was examined.

Daily dprime performance was calculated from the mean of all available n-back assessments within each day. Total daily mood was the summation of responses across the 3 questions presented during each assessment. Total step count from the iPhone and the Apple Watch was extracted for each day. Minimum, maximum, and mean daily heart rates for each day were obtained from the Apple Watch.

Summary measures for daily assessments were obtained for total daily mood, daily dprime, average heart rate, and total step count; means of all available daily assessments were calculated across the entire assessment period (6 weeks) and for individual weeks (1-6) to document change over the assessment period. No corrections for missing data and no other adjustments to raw data were made. Normality of all summary measures was assessed with visual examination of the data and with the Shapiro-Wilk test before further analysis.

#### Web-Based Full-Length Assessments Data Preparation and Cleaning

Absolute scores from validated self-report questionnaires were computed by summating responses within scales and providing summed scores for PHQ-9, PDQ-D, and UCLA-LS at each time point. To reduce multiple comparisons, overall scores from self-report questionnaires and CANTAB cognitive testing were calculated by taking the mean of outcome measures obtained at weeks 1, 3, and 6. This yielded overall means for SWM between errors, SWM strategy, RVP A′, and RVP median latency, as well as for self-report questionnaires (PHQ-9, PDQ-D, and UCLA-LS). Normality of data was assessed with visual examination of the data and with the Shapiro-Wilk test before further analysis.

#### Adherence Over Time

To examine whether the binary variable of adherence (response vs nonresponse) improved or declined over time, a series of logistic regression mixed models were carried out with study day (days 1−42) as a fixed factor and the participant as a random effect. Logistic regressions were also repeated separately for morning, afternoon, and evening n-back assessments to identify changes in response by time of day over the duration of the study.

Logistic regression models examined whether adherence to cognitive and mood assessments could be predicted by severity of depression symptoms at the onset of the study, as measured by the following covariates: PHQ-9, PDQ-D, and UCLA-LS scores from week 1. These included a covariate-by-day interaction term to examine variation by day. Assumptions of logistic regression models were investigated by examining the distribution and patterns of residuals versus fitted values.

To test whether adherence was associated with cognitive symptoms at study onset, a series of bivariate correlations (Pearson correlations or Spearman rank correlation, as appropriate) were completed. These explored the relationship between overall adherence with CANTAB cognitive measures at week 1. As this was an exploratory study, no corrections for multiple comparisons were made.

#### Daily Cognitive Assessment

Cognitive performance on the n-back was modeled using a longitudinal mixed-effects model with daily dprime as response variable, a fixed effect of study day, and a random effect of participant with random intercept and random slope. No covariates were examined. For the fixed-effect part of the model, we compared linear, quadratic, and cubic trends via likelihood ratio test and compared model parameters via maximum likelihood. This allowed the examination of different learning curves on the n-back to identify the best fit for change in performance over time. Each participant’s intercept (representing initial level of performance) and slope (representing learning rate) were extracted.

Summary n-back measures (mean, intercept, and slope) were correlated with overall means of CANTAB outcome measures and self-report questionnaires (PHQ-9, PDQ-D, and UCLA-LS). N-back slope was also correlated with the total number of n-back assessments completed over the study period, to examine the effects of practice on learning rate. Pearson or Spearman correlations were performed as appropriate.

#### Daily Mood Assessment

Multilevel reliability of the 3 mood items was examined using the multilevel.reliability command in the *Psych* package of *R* [44]. The package takes into consideration missing data by including components of variance derived from multilevel mixed modeling and examines multiple sources of variance for each score based on generalizability theory.

Average daily mood was modeled using a longitudinal mixed-effects model with total daily mood as response variable, a fixed effect of study day, and a random effect of participant with random intercept and random slope. No covariates were examined. For the fixed-effect part of the model, linear, quadratic, and cubic trends were compared via likelihood ratio test, and model parameters were compared via maximum likelihood, identifying the best fit for change in mood over time.

Overall means for daily mood assessment from the entire assessment period were correlated with overall means from full-length questionnaires and CANTAB assessments to investigate concurrent validity of daily mood assessments and the relationship between daily mood and full-length cognitive assessments. Parametric or nonparametric correlations were completed as appropriate.

#### Activity and Heart Rate Data

Total step count from the iPhone and the Apple Watch was extracted for each day. Means were calculated for the duration of the study (overall means) and for each study week. Minimum, maximum, and average daily heart rates were obtained from the Apple Watch, and the mean daily heart rate was calculated over the study duration (overall mean) and for each study week. The correlation between overall means for steps measured from the iPhone and the Apple Watch was examined.

### Ethics Approval

The study was reviewed and approved by the Proportionate Review Sub-Committee of the Wales Research Ethics Committee 6 at Swansea University (REC reference: 17/WA/0042) and performed in accordance with the current version of the Declaration of Helsinki. All participants provided written informed consent before enrollment.

## Results

### Participants

Of the 37 eligible participants, 30 were enrolled (19 women and 11 men). Participants were aged between 19 and 63 years (mean age 37.2 years; SD 10.4) and had been on their current medication for an average of 9.9 months (range 0.4−94.3 months; SD 9.5). Current medications included serotonin antagonist and reuptake inhibitor (n=1), serotonin and norepinephrine reuptake inhibitors (n=5), selective serotonin reuptake inhibitors (n=20), and tricyclic antidepressants (n=4). Mean depression symptom severity, measured by the PHQ-9, was 9.1 (range 5-15; SD 3.1).

### Adherence

Descriptive statistics for adherence across the duration of the study and by study week are shown in Table 1. Full adherence (100%, 42/42 days) was seen in 21 of 30 participants for cognitive assessment, 15 of 30 participants for mood testing, and 13 of 30 participants for activity assessment. Periods of low adherence tended to cluster temporally (Multimedia Appendix 2). Because of a technical issue on the final study day, the evening session was not administered, resulting in lower adherence on day 42. However, logistic mixed modeling showed no deterioration in adherence (ie, responding at least once daily) over time for assessments of mood, cognition, or activity. Logistic regression confirmed that self-reported depressive symptoms, assessed by the PHQ-9, PDQ-D, and UCLA-LS, were not associated with level of adherence in mood or cognitive assessments. Adherence was not significantly correlated with any CANTAB measures at week 1 (maximum *rho*=0.15; *P=*.44).

Participants completed a mean of 86.8% of all possible n-back assessments (range 50%-99%, 63-125 of 126 assessments). Rate of responding in the morning (84%) was lower than the afternoon (87%) and evening (89%; χ^2^_2_=12.9). Furthermore, although adherence (responding at least daily) remained high throughout the duration of the study, logistic regression confirmed modest reductions in individual assessments (morning, afternoon, and evening) over the study duration (morning: fixed-effects estimate=−0.03, *P*=.02; afternoon: fixed-effects estimate=−0.02, *P*<.001; evening: fixed-effects estimate=−0.08, *P*<.001).

**Table 1:** Percentage adherence for cognitive (n-back) and mood assessments and percentage of watch-wearing days (step count) completed over the duration of the study (overall) and broken down by week (week 1 to week 6). Adherence for cognitive and mood assessments defined as participants completing at least 1 full assessment per day. Watch-wearing days for step count defined as days with a minimum of 100 steps and heart rate recorded.

Measure		Participant adherence (%)						
		Overall	Week 1	Week 2	Week 3	Week 4	Week 5	Week 6
**n-back**								
	Mean (SD)	95.63 (9.28)	94.29 (19.35)	93.33 (18.28)	93.33 (20.8)	99.05 (5.22)	100 (0)	93.81 (17.06)
	Range	66.7-100	0-100	28.6-100	14.3-100	71.4-100	100	28.6-100
**Mood**								
	Mean (SD)	94.60 (9.73)	93.81 (19.38)	92.38 (20.46)	92.38 (22.11)	98.57 (5.75)	99.05 (3.63)	91.43 (20.75)
	Range	66.7-100	0-100	28.6-100	14.3-100	71.4-100	85.7-100	14.3-100
**Step count**								
	Mean (SD)	90.08 (17.4)	94.54 (14.99)	92.12 (18.15)	92.38 (20.11)	92.38 (19.03)	92.86 (17.09)	85.22 (24.29)
	Range	21-100	28-100	14-100	14-100	28-100	28-100	14-100

### Daily Cognitive Assessment

**Figure 2:**
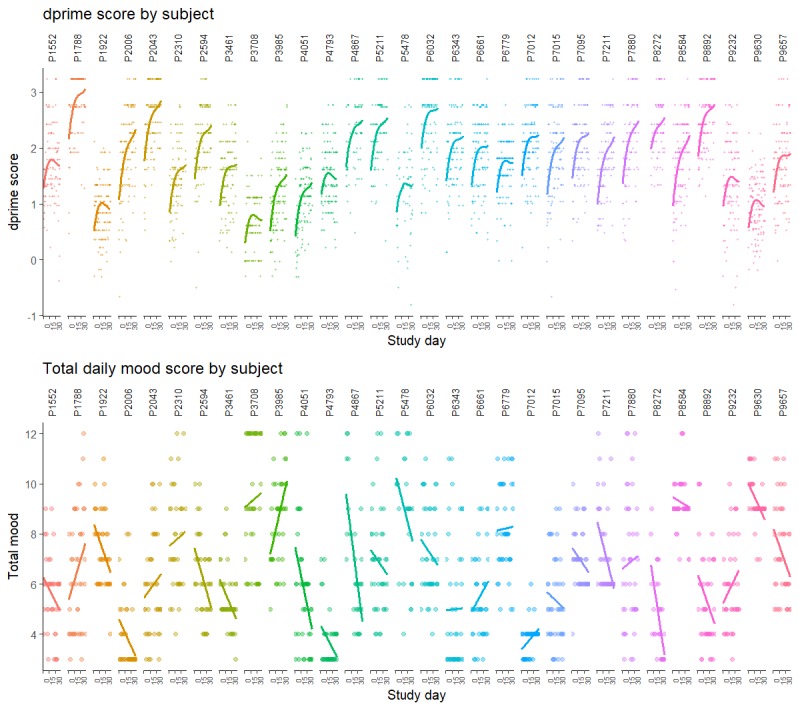
Trajectories in n-back performance and mood over time for study participants; each study day is represented on the x-axis. Top: Each dprime (up to 3 daily) is shown on the y-axis (higher scores denote better performance). Bottom: total mood is shown on the y-axis (higher scores denote more depressive symptoms).

Descriptive data for n-back assessments are presented in Table 2. Multilevel analysis of dprime score by study day confirmed a better fit for a cubic term rather than quadratic or linear models (Bayesian information criterion=1298.05; likelihood ratio=10.36; *P*=.001), indicating an initial rapid improvement in performance followed by a plateau. Model fits for each study participant are shown in Figure 2. Dprime slope showed no significant relationship with the number of n-back assessments completed (*rho*=−0.02, 95% CIs −0.37 to 0.34; *P*=.91).

Correlations between task performance metrics from the n-back and overall means from CANTAB cognitive assessments and self-report questionnaires were explored (Table 3). Participants with better performance on CANTAB showed higher intercept and better mean performance on the n-back. Depressive symptoms assessed with the PHQ-9 correlated with mean dprime, and correlations with dprime intercept approached but did not reach statistical significance (*P*=.06). No significant correlations were seen with PDQ-D or UCLA-LS, or dprime slope.

**Table 2:** Descriptive data for main outcome variables.

Outcome measure (daily)		Weeks assessed						
		Overall	Week 1	Week 2	Week 3	Week 4	Week 5	Week 6
**Daily dprime (n-back)**								
	Mean (SD)	1.8 (0.53)	1.38 (0.46)	1.72 (0.56)	1.79 (0.51)	1.92 (0.60)	1.97 (0.60)	1.96 (0.58)
	Range	0.7-2.8	0.4-2.2	0.6-3.0	0.6-2.84	0.71-3.01	0.84-3.01	0.56-2.88
**Total mood score**								
	Mean (SD)	6.54 (2.41)	6.96 (2.49)	6.75 (2.45)	6.54 (2.26)	6.60 (2.45)	6.15 (2.31)	6.26 (2.41)
	Range	3-12	3-12	3-12	3-12	3-12	3-12	3-12
**iPhone step count**								
	Mean (SD)	3762.81 (3168.59)	4124.48 (3610.95)	3605.83 (3207.43)	3961.29 (3282.68)	3751.95 (3305.45)	3404.67 (2438.33)	3745.64 (3053.46)
	Range	100-20,183	110-17,529	109-20,183	103-14,470	122-18,393	100-13,548	104-15,485
**Apple Watch step count**								
	Mean (SD)	6429.64 (4242.01)	6778.82 (4294.81)	6607.06 (4093.61)	6542.80 (4699.67)	6106.60 (3980.54)	6072.75 (4124.24)	6487.93 (4223.08)
	Range	125-22,360	133-19,560	144-21,533	448-22,360	125-19,808	155-22,130	293-20,049
**Heart rate, bpm^a^**								
	Overall mean (SD)	79.71 (10.47)	78.63 (8.72)	80.76 (11.98)	80.33 (12.87)	79.34 (9.65)	79.50 (8.75)	79.72 (10.17)
	Range	60-147	63-116	64-147	60-146	62-140	61-115	61-124

**^a^**bpm: beats per minute.

**Table 3:** Correlation coefficients (95% CIs) for daily cognitive assessments with full-length aggregate Cambridge Neuropsychological Test Automated Battery cognitive assessment outcome measures and full-length aggregate self-report questionnaires.

Outcome measures from full length assessment	n-back performance		
	Mean	Intercept	Slope
SWM^a^ between errors	−0.46^b^ (−0.70 to −0.11)	−0.51^b^ (−0.51 to −0.17)	−0.01 (−0.45 to 0.39)
SWM strategy	−0.37^c^ (−0.65 to −0.01)	−0.40^c^ (−0.67 to −0.04)	0.05 (−0.34 to 0.46)
RVP^d^ A'	0.50^b^ (0.17 to 0.73)	0.47^b^ (0.12 to 0.71)	0.29 (−0.10 to 0.58)
RVP median latency	−0.42^c^ (−0.73 to 0.001)	−0.46^c^ (−0.75 to −0.07)	−0.15 (−0.53 to 0.22)
Depressive symptoms (PHQ-9)^e^	−0.38^c^ (−0.65 to −0.01)	−0.36 (−0.65 to 0.01)	−0.18 (−0.51 to 0.24)
Cognitive problems (PDQ-D)^f^	−0.25 (−0.57 to 0.12)	−0.27 (−0.58 to 0.11)	0.04 (−0.37 to 0.32)
Loneliness (UCLA-LS)^g^	−0.29 (−0.59 to 0.09)	−0.26 (−0.57 to 0.12)	−0.21 (−0.54 to 0.18)

^a^SWM: spatial working memory.

^b^*P*≤.01.

^c^*P*≤.05.

^d^RVP: rapid visual information processing.

^e^PHQ-9: Patient Health Questionnaire-9.

^f^PDQ-D: Perceived Difficulties Questionnaire—Depression.

^g^UCLA-LS: University of California Los Angeles Loneliness Scale.

### Daily Mood Assessment

The 3 mood items showed overall good reliability indices, supporting the combined use of the 3 question items. Between-person reliabilities were high (R=0.97 averaged over time and with time nested within individuals), and within-person generalizability was moderate to high (R=0.75 for within-person variation with time nested within individuals).

Descriptive data for total mood are presented in Table 2. Multilevel analysis of total mood by study day confirmed the best fit for a linear model (Bayesian information criterion=73.38; likelihood ratio=6.14; *P*=.01). This model showed a modest overall linear improvement in mood over the course of the study (estimate of fixed effect of study day on mood=−0.0026, *P*=.01). However, there was a great deal of heterogeneity on mood trajectories over the study duration, as shown in model fits for each study participant in Figure 2.

Mean overall scores from daily mood assessments were correlated with full-length self-report questionnaires, showing moderate correlations (Table 4). Self-reported depression (PHQ-9) and cognitive symptoms (PDQ-D) correlated more highly with daily mood assessments than self-reported loneliness as measured by the UCLA-LS.

Significant correlations between dprime mean and intercept were seen for total mood scores, for question items assessing lack of interest, and for low mood (Table 4). Correlations between n-back performance and daily reported cognitive symptoms and all correlations with dprime slope were nonsignificant (*P=*.12-.79). Examining the relationship between daily mood assessment and CANTAB measures, SWM between errors and strategy showed moderate correlations with daily reported mood, whereas correlations with RVP outcome measures were nonsignificant.

**Table 4:** Correlation coefficients (95% CIs) for daily mood assessments with full-length self-report measures of depression, daily cognitive assessments, and full-length cognitive assessments on Cambridge Neuropsychological Test Automated Battery.

Outcome measures from full length assessments and daily cognitive assessments	Total mood score	Daily question items		
		Low mood (feeling down, depressed, hopeless)	Cognitive symptoms (trouble concentrating on things)	Lack of interest (lack of interest or pleasure)
Depressive symptoms (PHQ-9)^a^	0.69^b^ (0.44 to 0.84)	0.56^b^ (0.24 to 0.77)	0.69^b^ (0.44 to 0.85)	0.70^b^ (0.45 to 0.85)
Cognitive problems (PDQ-D)^c^	0.65^b^ (0.37 to 0.82)	0.50^b^ (0.16 to 0.73)	0.68^b^ (0.42 to 0.84)	0.64^b^ (0.36 to 0.82)
Loneliness (UCLA-LS)^d^	0.45^e^ (0.10 to 0.70)	0.47^b^ (0.13 to 0.72)	0.35 (−0.02 to −0.63)	0.45^e^ (0.10 to 0.70)
dprime mean	−0.41^e^ (−0.67 to −0.06)	−0.36^e^ (−0.64 to 0.00)	−0.28 (−0.58 to 0.08)	−0.52^b^ (−0.74 to −0.19)
dprime intercept	−0.42^e^ (−0.68 to −0.07)	−0.38^e^ (−0.65 to −0.03)	−0.29 (−0.59 to 0.08)	−0.52^b^ (−0.74 to −0.19)
dprime slope	−0.10 (−0.51 to 0.33)	−0.02 (−0.52 to 0.38)	−0.02 (−0.46 to 0.35)	−0.13 (−0.51 to 0.29)
SWM^f^ between errors	0.49^b^ (0.15 to 0.73)	0.52^b^ (0.19 to 0.74)	0.39^e^ (0.03 to 0.66)	0.49^b^ (0.15 to 0.73)
SWM strategy	0.44^e^ (0.09 to 0.70)	0.43^e^ (0.07 to 0.69)	0.41^e^ (0.05 to 0.68)	0.41^e^ (0.05 to 0.68)
RVP^g^ A'	−0.14 (−0.48 to 0.24)	−0.06 (−0.41 to 0.32)	−0.13 (−0.47 to 0.25)	−0.20 (−0.53 to 0.18)
RVP median latency	0.23 (−0.18 to 0.58)	0.27 (−0.09 to 0.57)	0.18 (−0.19 to 0.51)	0.28 (−0.11 to 0.61)

^a^PHQ-9: Patient Health Questionnaire-9.

^b^*P*≤.01.

^c^PDQ-D: Perceived Difficulties Questionnaire—Depression.

^d^UCLA-LS: University of California Los Angeles Loneliness Scale.

^e^*P*≤.05.

^f^SWM: spatial working memory.

^g^RVP: rapid visual information processing.

### Activity and Heart Rate

**Figure 3:**
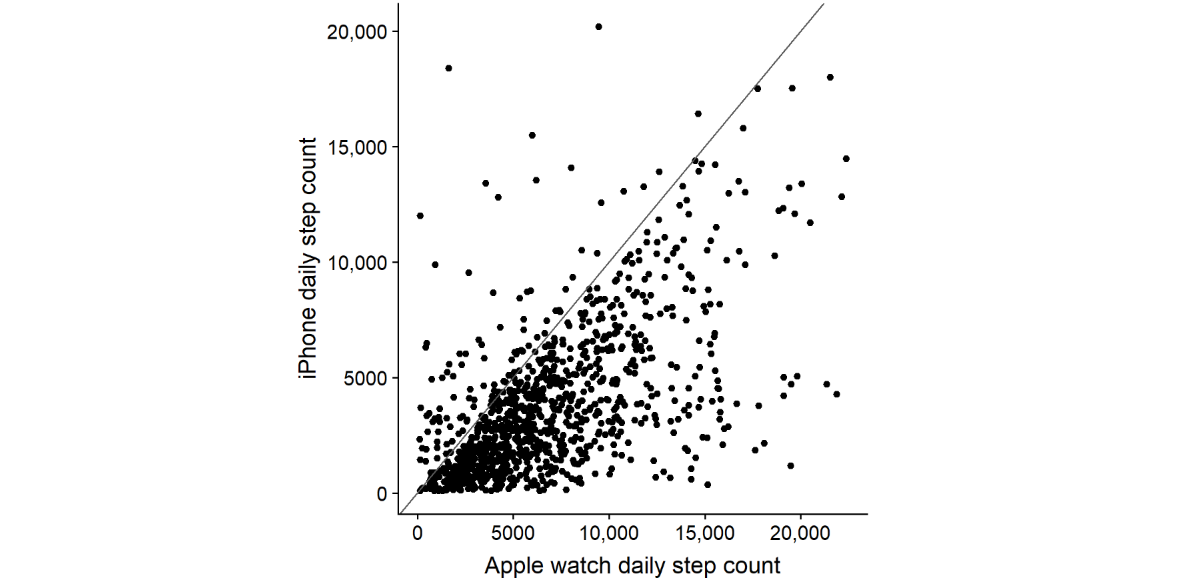
Scatter plot of mean daily step count as measured by the Apple Watch vs. the iPhone, and reference line for perfect agreement between devices.

Descriptive statistics for step counts and heart rate are presented in Table 2. A moderate correlation was seen between step counts registered on the 2 devices (*rho=*0*.*61; 95% CI 0.57-0.65; *P*<.001), but there were also instances of marked discrepancy (Figure 3). Overall, the Apple Watch provided a higher step count estimate than the iPhone. Measurement issues were noted for heart rate using the Apple Watch, with individual heart rates registered including a minimum of 22 beats per minute, which was not biologically plausible.

## Discussion

### Principal Findings

This study demonstrated the feasibility of daily assessments of cognition and mood in mild-to-moderate MDD. The study spanned 6 weeks, corresponding to the time during which response to antidepressant pharmacotherapy efficacy would expect to be demonstrated, indicating that high levels of adherence can be achieved and retained over this time frame.

Exploratory analyses examined the relationship between high-frequency mood and cognitive assessment and validated full-length cognitive assessments and questionnaires. These analyses aimed to establish the degree to which brief frequent assessments capture similar information to validated cognitive assessments and rating scales. Daily mood assessments showed moderate to strong correlations with validated self-report questionnaires of depression, cognitive problems, and loneliness. Correlations were highest for the PHQ-9, a scale designed as both a diagnostic instrument and a severity measure [34], which also showed the highest item overlap with high-frequency assessments. Daily n-back performance correlated moderately with performance on standardized tests of working memory and sustained attention. Findings support the concurrent validity of the measures examined during daily assessments.

### Adherence

Adherence, defined as engaging with cognitive and mood assessments at least once daily, was very high (95%-96%), did not deteriorate over time, and was not predicted by depressive symptoms or cognitive function at study onset. These adherence rates, as well as the overall rate of responding to high-frequency assessments in the current study (≈87% for all possible cognitive assessments), are in keeping with previous compliance rates reported in high-frequency assessments in psychopharmacology, around 50% to 90% [45]. However, it is notable that although this study was significantly longer in duration than most previous high-frequency assessment studies, spanning 6 weeks rather than the typical 1- to 2-week duration, the daily frequency of assessment was lower, with most other studies typically sampling 5 to 10 times per day [45]. Previous studies in patients with mood disorders have shown good overall feasibility and acceptability of high-frequency assessments, although there is likely to be an interaction between protocol burden and burden of illness [46]. The brevity of the current protocol in conjunction with the proximity to wearable assessments may have helped to support the high levels of compliance seen here.

Participants reported that completing assessments was easier when study sessions fit into their daily routines, and that periods of high and low mood affected their motivation to complete assessments. Adherence was also affected by technical problems for some participants, and by forgetting to wear the Apple Watch because of low mood or bereavement. Study center support and reminders during nonadherent periods provided a framework to enable participants to maintain a high level of engagement with the study.

### Change Over Time

Participants’ performance on the n-back improved over time. Overall, mood symptoms showed a modest concurrent improvement, albeit with great heterogeneity in the trajectories observed over the assessment period. Participants were stabilized on monotherapy at the time of assessment, and many had started their current treatment many months before study participation (9.9 months on average). Improvements on the n-back, therefore, likely reflect the influence of practice effects and task specialization. Participants reported continued improvement in task performance as a motivator for engagement. This finding is supported by studies exploring gamification of tasks, where the use of game design elements (eg, points and scoreboards) can improve motivation [47,48].

Importantly, very few participants reached and maintained ceiling levels of performance on the n-back. The symbols presented were designed to be hard to name, and each testing occasion drew 9 items from a stimulus pool of 227 items. Almost all participants felt that the task was challenging yet achievable. Attainability encouraged them to set personal goals to improve or maintain their scores, indicating that striking a balance between difficulty and attainability can promote engagement [49].

Individual learning rates for each participant were reflected in their n-back slope, which did not correlate significantly with either CANTAB cognitive test measures or self-reported mood. This suggests that the capacity to improve performance is not directly affected by either depressive symptoms or cognitive impairment, consistent with research in a previous study showing that practice effects in cognitive tasks were not moderated by depressive symptomatology [50].

### Association Between Measures

The n-back paradigm is commonly used alongside functional neuroimaging, where it activates a network of frontoparietal areas [51]. Research suggests that n-back is not simply a measure of working memory capacity but depends on functions such as updating, inhibition, and attention [52]. Consistent with this suggestion, n-back mean and intercept correlated with full-length CANTAB cognitive tests of attention and working memory, supporting the use of n-back performance as a sensitive but nonspecific marker of cognitive function.

The trajectory of moods reported by patients during the course of the study was highly heterogeneous, showing no clear relationship with change in cognitive performance (Figure 2). However, we observed a significant association between aggregate daily mood measures with cognitive measures from CANTAB and n-back task performance (mean and intercept).

### Relationship With Full-Length Assessments

The relationship between self-report questionnaires and high-frequency assessments of symptoms has been examined in a number of clinical conditions. Although in some cases the correlations are good [53], there can be a mismatch, with questionnaires relying on retrospective recall tending to overstate the severity and frequency of symptoms [54]. Retrospective recall shows distortion in favor of more salient or unique events at the expense of the more mundane [54], and depression is associated with negative biases in recollection during periods of low mood [55]. High-frequency assessment may be particularly useful in patients with MDD for ensuring accurate recording of the course of their illness and treatment response.

In this study, correlations between daily measures and validated self-report questionnaires were moderate to high. Discrepancies between objectively and subjectively assessed cognitive function have been reported before, with the latter being affected by depressed mood [15,24,25]. Our results confirm this association. PDQ-D scores were correlated with daily mood assessments but not with cognitive performance, indicating that self-reported cognitive function cannot substitute for objective assessments.

### Limitations

As our study focused on patients with mild-to-moderate MDD who volunteered for participation, it is unclear whether results would generalize to patients with different severity or to those who are less motivated. In addition, assessment using a small touch screen may not be feasible for patients with visual impairments or those requiring a larger typeface.

Step counts collected via the Apple Watch and the iPhone were discrepant, which could be accounted for by differences in wearing patterns but undermines the reliability of activity data from either device. Measurement issues with heart rate data may reflect that the equipment was not of medical grade, or occasions when the Apple Watch was not fitted sufficiently tightly for reliable measures to be obtained. Although the wearable nature and ease of use of the technology allow for data to be collected over longer periods of time, our findings indicate that caution is required when this equipment is used to examine heart rate in scientific research. Variable accuracy for wrist-worn heart rate monitors, including the Apple Watch, compared with electrocardiogram measurement has also been noted previously in brief comparisons of bouts of exercise [56].

### Conclusions

This study supports the feasibility and validity of high-frequency assessment on wearable devices to assess cognitive function and mood in patients with MDD. The study spanned 6 weeks, indicating that high levels of adherence can be achieved and retained over this time frame. Our study suggests that these methods can be used to monitor cognitive function and mood symptoms after the initiation of treatment for depression.

## Appendix

Multimedia Appendix 1 Discussion guides for semistructured interviews at study onset and end.

Multimedia Appendix 2 Periods of adherence for cognition, mood, and activity assessment.

## Abbreviations

CANTAB: Cambridge Automated Neuropsychological Test Battery
MDD: major depressive disorder
PDQ-D: Perceived Difficulties Questionnaire in Depression
PHQ-9: Patient Health Questionnaire-9
RVP: rapid visual information processing
SWM: spatial working memory
UCLA-LS: University of California Los Angeles Loneliness Scale
