# Supplementary material for: Wearable Technology for High-Frequency Cognitive and Mood Assessment in Major Depressive Disorder: Longitudinal Observational Study
Source: JMIR Ment Health. 2019 Nov 18;6(11):e12814. doi: 10.2196/12814 (PMC6887827; doi:10.2196/12814)
Supplement: Multimedia Appendix 1 [file mental_v6i11e12814_app1.pdf]

Multimedia Appendix 1

First Study Visit - Interview Discussion Guide

Introduction
Before we go into the details of the study I'd like to ask you a few questions to get an understanding of your expectations and interest in taking part.
We're really interested in your personal experience and perspective. So there are no wrong or right answers, please do feel free to be honest and open.

1. Can you talk me through why you were interested in taking part in this study?
tease out interest in contributing to research / cognition / mood / device
probe on incentive as motivation

2. What do you understand from the idea of 'cognitive testing' in general?

3. Have you used any technology to track mood, activity or anything else before? If yes:
which ones did you use?
how often do you use them?
what are the factors that you think influence how often you use them?
probe on general technology and device use.

If not, why not:

4. What are your expectations for what it will be like to do cognitive and mood tasks every day for 6 weeks?
explore if they feel it will be a challenge
explore what barriers or enablers they foresee

5. How do you feel about doing these tasks on an Apple Watch, which you'll be wearing every day for the next 6 weeks?
explore any specific expectations related to the device
explore any specific barriers or enablers related to the device

6. How did you find doing the Two-Back task [*demo*]?

7. Questions asked - Note down any questions asked throughout the session

8. Dates offline or abroad

Final Study Visit - Interview Discussion Guide

Introduction
Thank you again for taking part in the Cognition and Mood Study.
We're really grateful for your involvement.
This conversation is to learn about your experience doing the daily cognitive and mood tasks so we can understand how to better assess cognition in everyday life.
No right or wrong answers; looking for honest and open feedback.
No right or wrong way of interacting; do as feels most natural to you.
Conversation will take 90 mins. Tell me if that's ok, if you need to stop or break.

1. We didn't get the chance last time to have a proper introduction, so to start I wonder if you can just tell me a bit more about yourself?
Lifestyle / routines.
Typical day.
Show across places / objects in the house - get up and walk around if appropriate.

Has anything in your normal routines changed over the past couple of months?
Any external events / factors.
Across work / hobbies / family / friends.

2. General experience with daily tasks over time
Can you tell me how you got along overall with completing the cognitive and mood tasks everyday?
How was it different to what you expected?
Over the past 6 weeks were there any changes in your experience of doing the tasks?
Study schedule timeline.
Experience at the start / once familiar / over time / during difficult moments.
Explore changes in compliance and motivation.
Explore contextual factors across each.

3. Moments around motivation and compliance
Can you tell me more about the moments when it was difficult to complete the cognitive and mood assessments?
Barriers.
Across contexts appropriate/inappropriate.
Factors that impact performance.

Can you talk me through the moments when you were happy and it was easy to do the cognitive and affect assessments?
Exploring what factors supported compliance, motivation.
Exploring across contexts / environments / places / people involved.

What do you think kept you interested in going back to the app and completing the assessments?
Exploring what specific factors supported compliance, motivation.
Exploring across contexts / environments / places / people involved.

To what extent do you think the Apple Watch device impacted your ability and interest in doing the daily cognition and mood tasks?

4. Detail around tasks
Can you tell me how you felt completing the two-back tasks?
Ease vs difficulty.
Interest and level of motivation.
Level of understanding.

Can you tell me how you felt about the feedback you were given after the two-back tasks?
Ease vs difficulty.
Interest in information.
Level of understanding.

Can you tell me how you felt about answering the mood questions at the end of the day?
Ease vs difficulty.
Interest and level of motivation.
Level of understanding.

Let's go to the Study Summary screen. Can you show it to me? Can you tell me how you felt about the information on this screen?
For each piece of information:
- Level of understanding.
- Interest in information.
- Impact on motivation.

5. CANTAB
Can you describe your experience of doing the CANTAB tasks?
Ease vs difficulty.
Interest and level of motivation.
Level of understanding.

To what extent do you think doing the CANTAB tasks affected the rest of your experience of completing the daily cognitive and mood tasks on your watch?

6. Report
<u>Either:</u> Yesterday you were sent the end of study report. What did you think about it?
<u>Or:</u> Here is your end of study report. Can you think aloud as you read through it?
Can you tell me what you understand this data is telling you? Does this sound right to you?
Explore level of understanding.
Explore level of matching expectations.

What else would you be interested in seeing in your report?
Explore across data points.
Explore physical / psychological / social / environmental measures.

To what extent do you feel that knowing about the fact that you would get a report have an impact on your taking part over the past couple of weeks?
Explore impact on compliance and motivation.
Explore any feedback expected.

7. Set-up and support

To what extent did you feel that the initial set-up at our offices gave you enough support before starting the study?

If study centre used
You seemed to use the study centre [number of times and means used]? Can you tell me about that experience?

If study centre not used
You did not contact the study centre? Can you tell me why not?
What do you think would have been the circumstances in which you would have been in touch?
Explore any barriers in study centre set-up.
Explore across different means of contact.

To what extent did you use the study handbook?
Explore when used, why.
Explore level to which it was helpful or information was missing.

8. Long term
How would you feel about continuing to use the cognition and mood study app for longer?
What would keep you interested in taking part over that time?
How did you feel about your data being used to assess your cognition?

Do you have any final questions or comments for me?
