# Supplementary figures and images for: Wearable Technology for High-Frequency Cognitive and Mood Assessment in Major Depressive Disorder: Longitudinal Observational Study
Source: JMIR Ment Health. 2019 Nov 18;6(11):e12814. doi: 10.2196/12814 (PMC6887827; doi:10.2196/12814)

## Slide 1
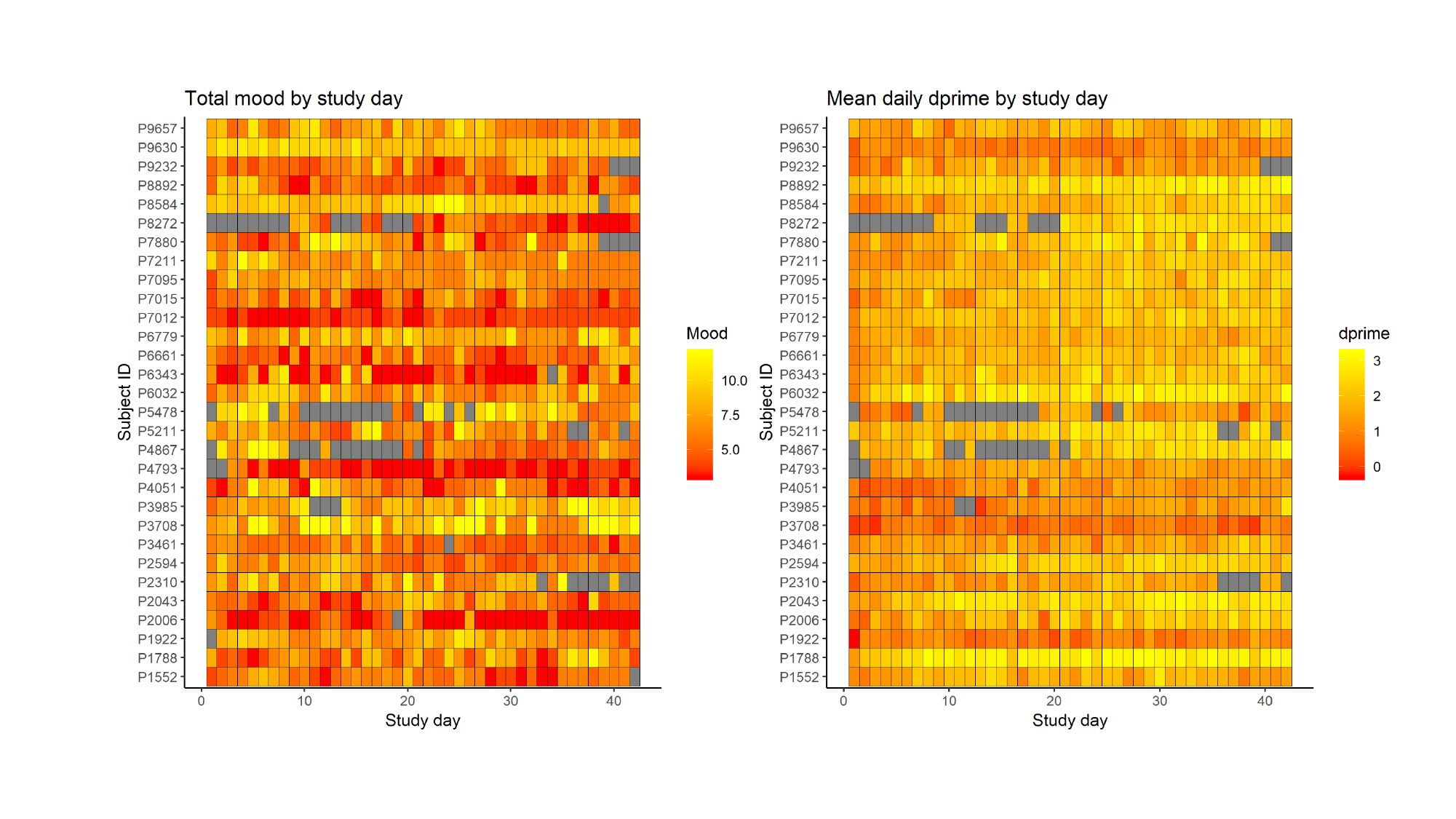

## Slide 2
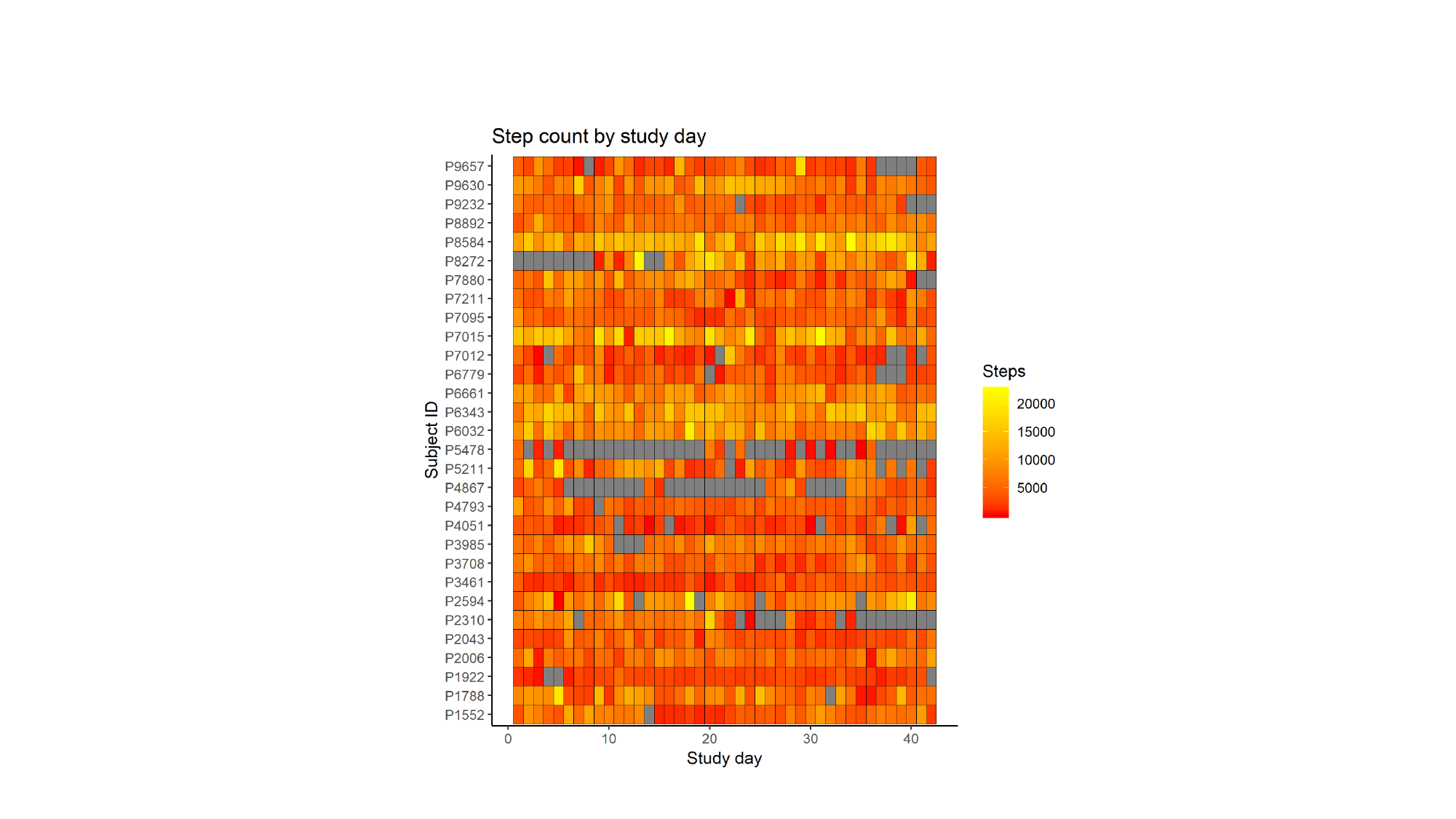

Supplement: Multimedia Appendix 2 [file mental_v6i11e12814_app2.pptx]
